# Supplementary figures and images for: SARS-CoV-2 Delta (B.1.617.2) variant replicates and induces syncytia formation in human induced pluripotent stem cell-derived macrophages
Source: PeerJ. 2023 Mar 2;11:e14918. doi: 10.7717/peerj.14918 (PMC9985896; doi:10.7717/peerj.14918)

**Delta**

**Omicron**

**Mock**

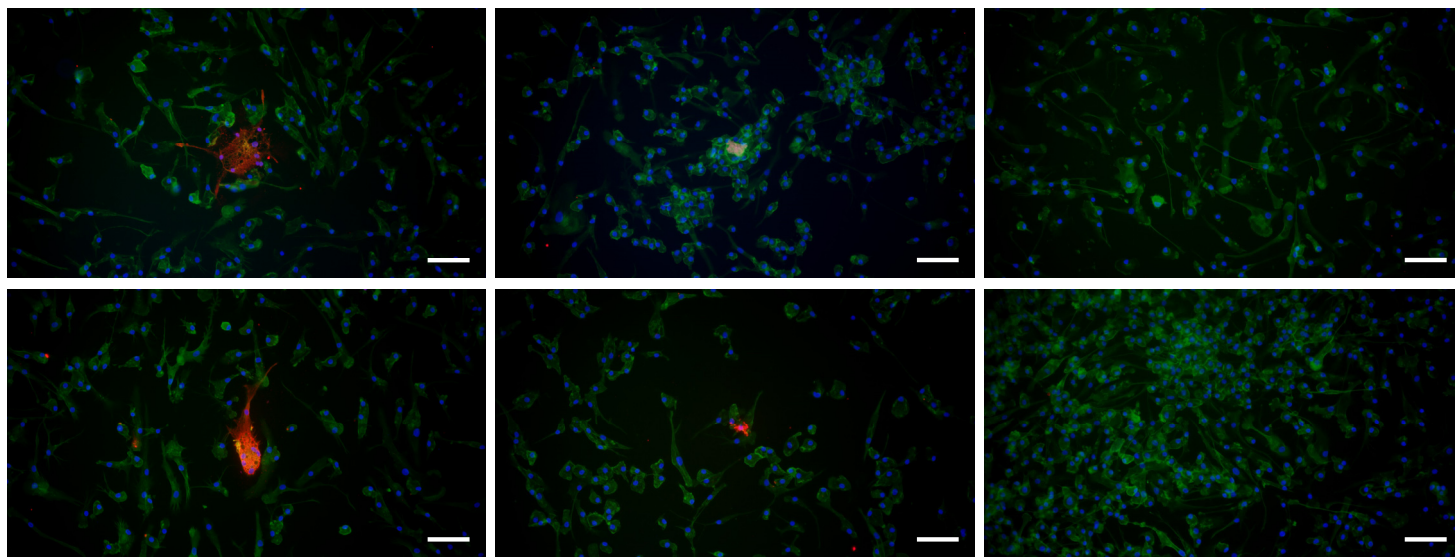

**Figure S1: Delta and Omicron variants infect iMΦ.**

Supplement: Supplemental Information 1 — Fluorescence microscopy of iMΦ infected with SARS-CoV-2 Delta and Omicron variants (MOI =0.1). Cells were adsorbed with virus for 2 h and fixed at 72 hpi. Cells were stained with primary anti-SARS-CoV-2 N antibody followed by secondary Alexa Fluor 568-conjugated anti-mouse IgG antibody (red) and Alexa Fluor 488-conjugated phalloidin (green). Cell nuclei were counterstained with DAPI (blue). Scale bars, 50 µm. [file peerj-11-14918-s001.pdf]

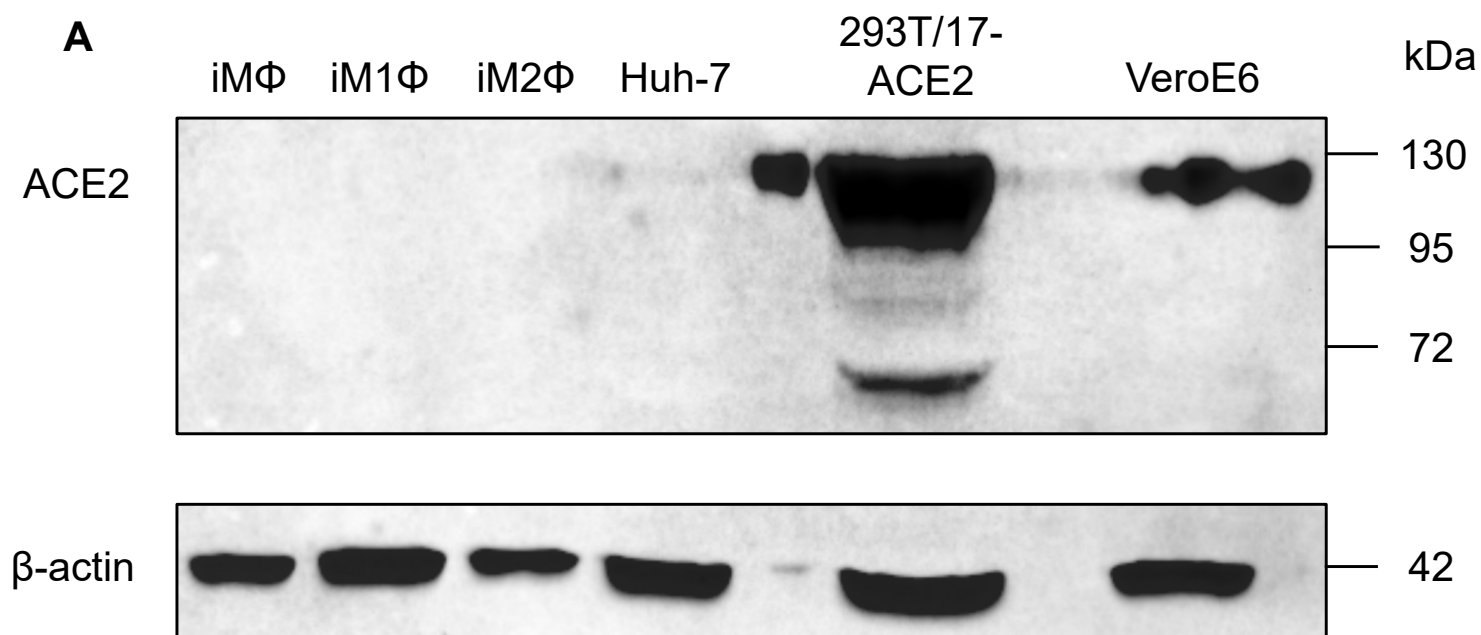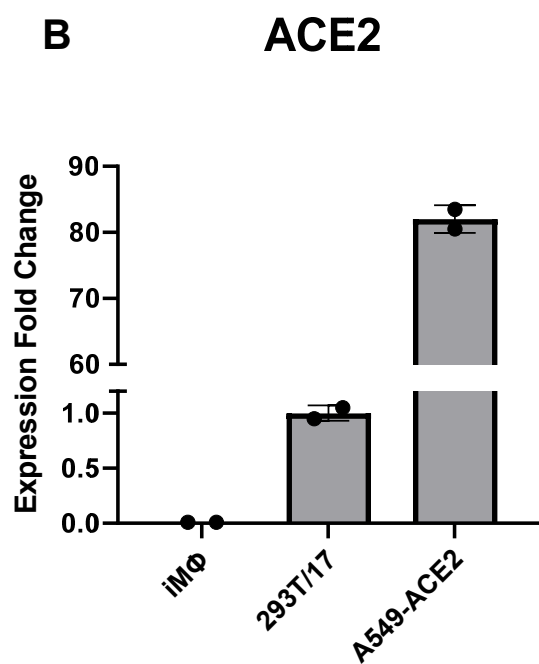

**Figure S2: iMΦ have undetectable expression of ACE2.**

Supplement: Supplemental Information 2 — (A) Western blot analysis of ACE2 protein expression in naïve iMΦ , M1-phenotype iMΦ (iM1 Φ ), M2-phenotype iMΦ (iM2 Φ), Huh-7, 293T/17-ACE2 and VeroE6 cells. 293T/17-ACE2 cells were stably transfected to express codon-optimized ACE2. (B) Bar graph displays the relative gene expression fold change of ACE2 mRNAs in iMΦ , 293T/17, and A549-ACE2 cells. A549-ACE2 cells were stably transfected to express native ACE2. Total RNA was extracted from cell lysates. Gene expressions were quantified in duplicate by RT-qPCR. Data are expressed as fold change in ACE2 gene expression compared with 293T/17 cells after normalization to GAPDH using 2−ΔΔCT method. Data were shown as means ± SD (n = 2 in each group). [file peerj-11-14918-s002.pdf]

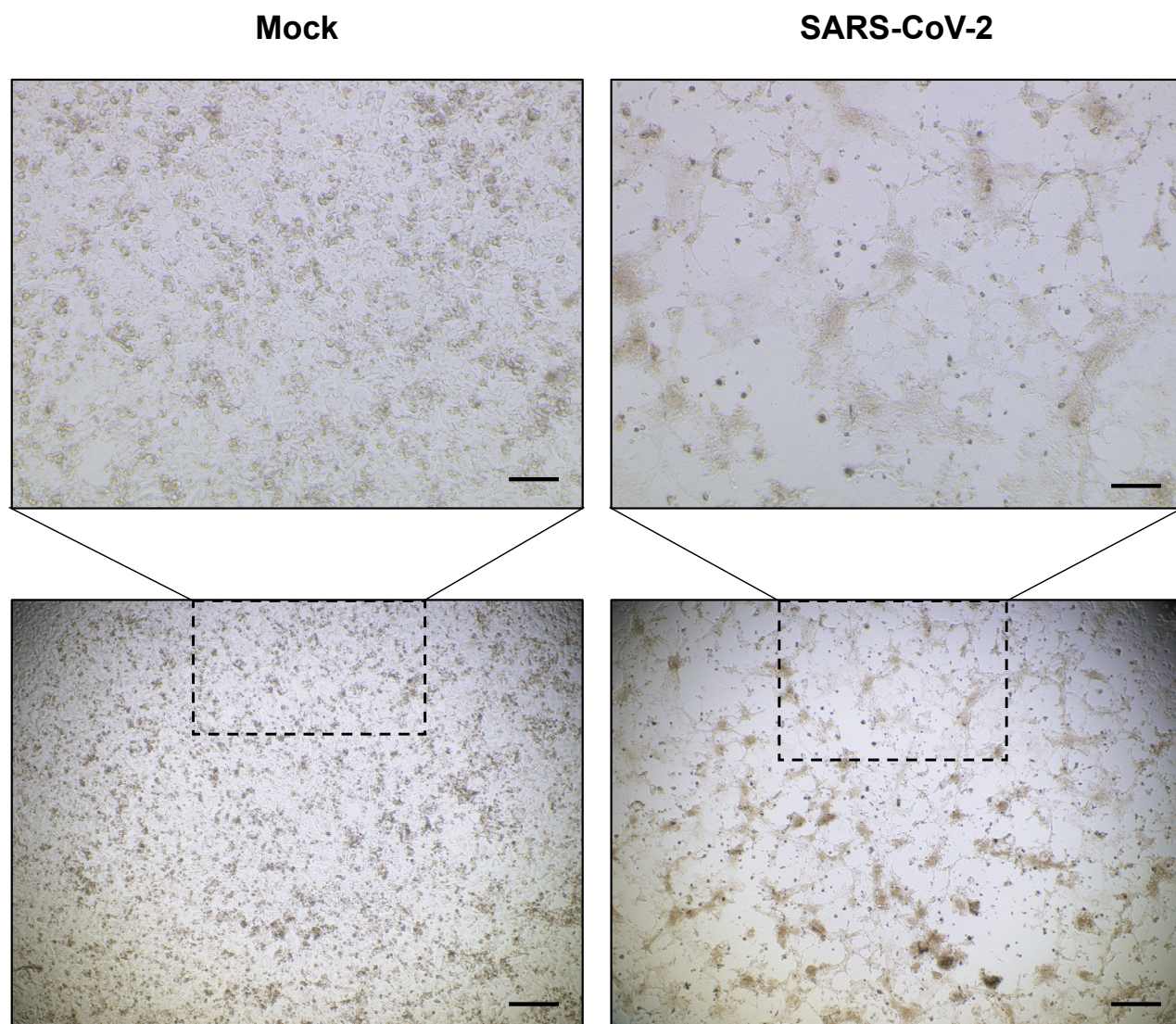

**Figure S3: A549-ACE2 cells show cytopathic effects after SARS-CoV-2 infection.**

Supplement: Supplemental Information 3 — Representative bright-field microscopy of A549-ACE2 cells infected with SARS-CoV-2. Cells were incubated with serially diluted virus samples for 72 hrs and then were examined for cytopathic effects (CPEs) to determine viral titers using the TCID50 assay. Mock infection was used as a control. Scale bars, 100 µm (above pictures), 200 µm (below pictures). [file peerj-11-14918-s003.pdf]

## CD86

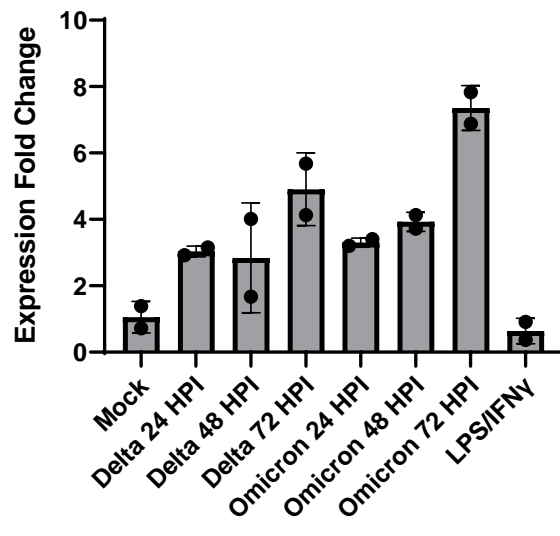

**Figure S4: SARS-CoV-2 infection of iM $\Phi$  activates their CD86 gene expression.**

Supplement: Supplemental Information 4 — Bar graphs display the relative gene expression fold change of CD86 mRNAs in iMΦ infected with SARS-CoV-2 Delta or Omicron variant (MOI =0.1) and iMΦ pre-treated with lipopolysaccharides (LPS) and interferon-gamma (IFN-γ). Total RNA was extracted from cell lysates at 24 h after LPS/IFN- γ treatment or at 24, 48, and 72 hpi for SARS-CoV-2 infection. Gene expressions were quantified in duplicate by RT-qPCR. Data are expressed as fold change in CD86 gene expression compared with mock infection after normalization to GAPDH using 2−ΔΔCT method. Data were shown as means ± SD (n = 2 in each group). [file peerj-11-14918-s004.pdf]

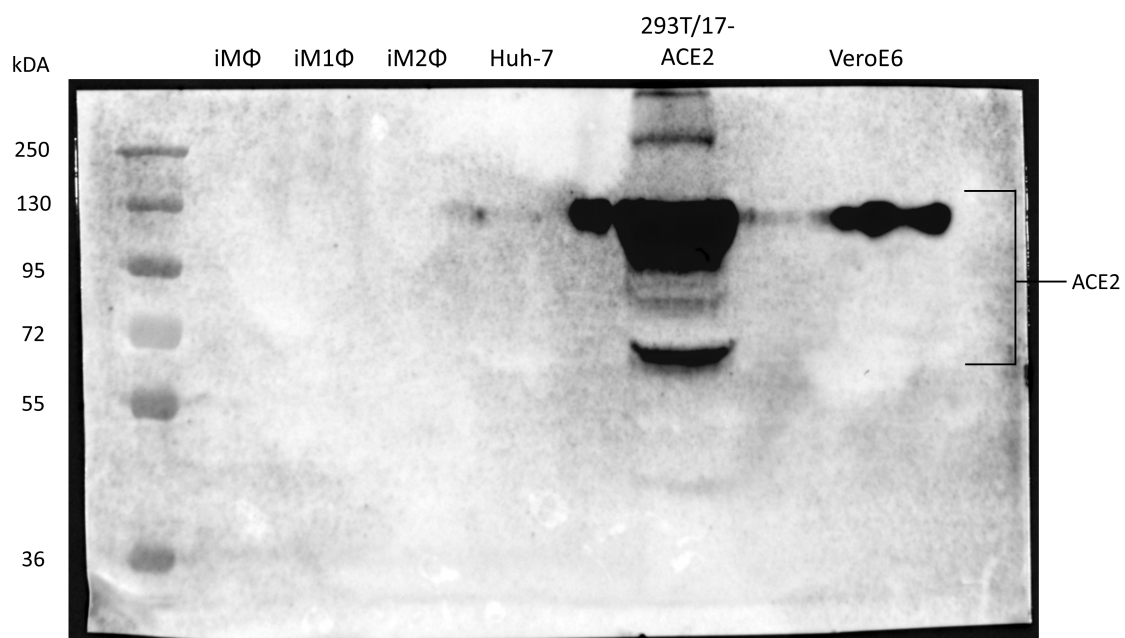

Supplement: Supplemental Information 10 [file peerj-11-14918-s010.pdf]

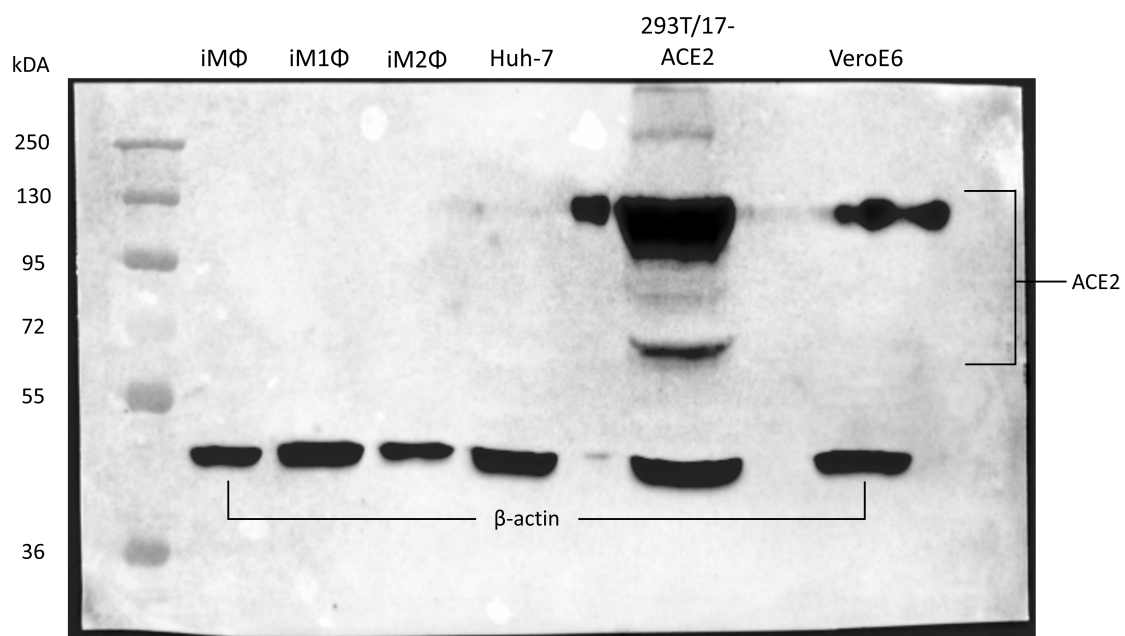

Supplement: Supplemental Information 11 [file peerj-11-14918-s011.pdf]
