# Supplementary material for: SARS-CoV-2 Delta (B.1.617.2) variant replicates and induces syncytia formation in human induced pluripotent stem cell-derived macrophages
Source: PeerJ. 2023 Mar 2;11:e14918. doi: 10.7717/peerj.14918 (PMC9985896; doi:10.7717/peerj.14918)
Supplement: Supplemental Information 5 [file peerj-11-14918-s005.docx]

**Table S1: Primers for qRT-PCR.**

| **Name** | **Forward Primer** | **Reverse Primer** | **Reference** |
| --- | --- | --- | --- |
| ACE2 | GGGATCAGAGATCGGAAGAAGAAA | AGGAGGTCTGAACATCATCAGTG | (Ma et al., 2020) |
| CD86 | CTGCTCATCTATACACGGTTACC | GGAAACGTCGTACAGTTCTGTG | (Mikhalkevich et al., 2021) |
| IL-1β | AAGCTGATGGCCCTAAACAG | AGGTGCATCGTGCACATAAG |  |
| IL-6 | CCAGCTATGAACTCCTTCTC | GCTTGTTCCTCACATCTCTC |  |
| IL-8 | TTTTGCCAAGGAGTGCTAAAGA | AACCCTCTGCACCCAGTTTTC |  |
| IL-18 | TCTTCATTGACCAAGGAAATCGG | TCCGGGGTGCATTATCTCTAC |  |
| TNF-α | ATGAGCACTGAAAGCATGATCC | GAGGGCTGATTAGAGAGAGGTC |  |
| CCL2 | GAGAGGCTGAGACTAACCCAGA | ATCACAGCTTCTTTGGGACACT |  |
| IFN-α | GACTCCATCTTGGCTGTGA | TGATTTCTGCTCTGACAACCT |  |
| GAPDH | TGCACCACCAACTGCTTAGC | GGCATGGACTGTGGTCATGAG |  |

**References**

Ma D, Chen C-B, Jhanji V, Xu C, Yuan X-L, Liang J-J, Huang Y, Cen L-P, Ng TK. 2020. Expression of SARS-CoV-2 receptor ACE2 and TMPRSS2 in human primary conjunctival and pterygium cell lines and in mouse cornea. *Eye* 34:1212–1219. DOI: 10.1038/s41433-020-0939-4.

Mikhalkevich N, O’Carroll IP, Tkavc R, Lund K, Sukumar G, Dalgard CL, Johnson KR, Li W, Wang T, Nath A, Iordanskiy S. 2021. Response of human macrophages to gamma radiation is mediated via expression of endogenous retroviruses. *PLOS Pathogens* 17:e1009305. DOI: 10.1371/journal.ppat.1009305.
