# Supplementary material for: SARS-CoV-2 Delta (B.1.617.2) variant replicates and induces syncytia formation in human induced pluripotent stem cell-derived macrophages
Source: PeerJ. 2023 Mar 2;11:e14918. doi: 10.7717/peerj.14918 (PMC9985896; doi:10.7717/peerj.14918)
Supplement: Supplemental Information 6 [file peerj-11-14918-s006.docx]

**Table S2: Relative gene expression fold change of ACE2 mRNAs in different cell types.**

| **Group** | **Expression Fold Change of ACE2** | |
| --- | --- | --- |
| iMΦ | 0.01 | 0.01 |
| 293T/17 | 1.05 | 0.95 |
| A549-ACE2 | 80.53 | 83.49 |
