# Supplementary material for: SARS-CoV-2 Delta (B.1.617.2) variant replicates and induces syncytia formation in human induced pluripotent stem cell-derived macrophages
Source: PeerJ. 2023 Mar 2;11:e14918. doi: 10.7717/peerj.14918 (PMC9985896; doi:10.7717/peerj.14918)
Supplement: Supplemental Information 7 [file peerj-11-14918-s007.docx]

**Table S3: Viral titers in the cell supernatants of Delta- or Omicron-infected iMΦ at different hours post-infection.**

| **Hours post-infection** | **Viral titer (TCID_50_/mL)** | | | | | |
| --- | --- | --- | --- | --- | --- | --- |
|  | Delta (B.1.617.2) | | | Omicron (B.1.1.529) | | |
| 0 | 69.3 | 69.3 | 123.2348 | 0 | 0 | 0 |
| 24 | 389.7025 | 519.6767 | 164.3362 | 0 | 0 | 0 |
| 48 | 1232.348 | 924.1304 | 292.2357 | 0 | 0 | 0 |
| 72 | 51.96767 | 29.22357 | 29.22357 | 0 | 0 | 0 |
