# Supplementary material for: SARS-CoV-2 Delta (B.1.617.2) variant replicates and induces syncytia formation in human induced pluripotent stem cell-derived macrophages
Source: PeerJ. 2023 Mar 2;11:e14918. doi: 10.7717/peerj.14918 (PMC9985896; doi:10.7717/peerj.14918)
Supplement: Supplemental Information 8 [file peerj-11-14918-s008.docx]

**Table S4: Relative gene expression fold change of cytokine mRNAs in different** **iMΦ groups.**

| **Group** | **Expression Fold Change** | | | | | | | | | | | |
| --- | --- | --- | --- | --- | --- | --- | --- | --- | --- | --- | --- | --- |
|  | IL-1β | | | IL-6 | | | IL-8 | | | IL-18 | | |
| Mock | 1.04 | 0.77 | 1.25 | 0.80 | 0.63 | 1.98 | 1.08 | 0.79 | 1.18 | 1.24 | 0.72 | 1.12 |
| Delta  24 HPI | 0.70 | 0.65 | 0.72 | 0.66 | 0.65 | 0.62 | 0.31 | 0.35 | 0.36 | 0.81 | 0.96 | 1.06 |
| Delta  48 HPI | 1.03 | 0.75 | 0.92 | 0.34 | 0.29 | 0.37 | 0.23 | 0.25 | 0.31 | 1.85 | 1.65 | 0.87 |
| Delta  72 HPI | 2.75 | 2.45 | 1.67 | 3.90 | 3.99 | 0.72 | 0.50 | 0.53 | 0.31 | 3.61 | 3.45 | 2.29 |
| Omicron 24 HPI | 0.73 | 0.65 | 0.81 | 0.59 | 0.68 | 0.86 | 1.26 | 1.14 | 1.48 | 1.17 | 1.10 | 1.45 |
| Omicron 48 HPI | 0.45 | 0.47 | 0.53 | 0.37 | 0.32 | 0.39 | 0.30 | 0.25 | 0.35 | 1.45 | 1.32 | 1.68 |
| Omicron 72 HPI | 2.16 | 1.81 | 0.99 | 3.63 | 4.08 | 1.05 | 0.72 | 0.56 | 0.40 | 5.85 | 4.76 | 3.59 |
| LPS/IFNγ | 18.10 | 12.82 | 8.37 | 82.54 | 80.38 | 55.26 | 2.06 | 1.38 | 1.44 | 3.52 | 2.22 | 1.88 |

| **Group** | **Expression Fold Change** | | | | | | | | |
| --- | --- | --- | --- | --- | --- | --- | --- | --- | --- |
|  | TNF-α | | | CCL2 | | | IFN-α | | |
| Mock | 0.53 | 0.28 | 6.74 | 0.94 | 0.93 | 1.14 | 1.11 | 0.79 | 1.14 |
| Delta  24 HPI | 0.57 | 0.44 | 0.50 | 0.26 | 0.23 | 0.23 | 0.20 | 0.20 | 0.21 |
| Delta  48 HPI | 1.24 | 1.24 | 1.36 | 0.35 | 0.26 | 0.34 | 0.53 | 0.58 | 0.51 |
| Delta  72 HPI | 0.39 | 0.43 | 1.05 | 0.99 | 0.92 | 1.23 | 0.71 | 0.61 | 0.41 |
| Omicron 24 HPI | 1.18 | 1.06 | 0.77 | 0.26 | 0.26 | 0.53 | 0.47 | 0.46 | 0.36 |
| Omicron 48 HPI | 0.73 | 0.79 | 0.90 | 0.44 | 0.29 | 0.59 | 0.59 | 0.62 | 0.65 |
| Omicron 72 HPI | 0.38 | 0.46 | 0.86 | 0.57 | 0.68 | 0.60 | 1.37 | 0.78 | 0.57 |
| LPS/IFNγ | 9.72 | 9.26 | 3.30 | 3.01 | 2.70 | 3.02 | 0.18 | 0.13 | 0.12 |
