# Supplementary material for: SARS-CoV-2 Delta (B.1.617.2) variant replicates and induces syncytia formation in human induced pluripotent stem cell-derived macrophages
Source: PeerJ. 2023 Mar 2;11:e14918. doi: 10.7717/peerj.14918 (PMC9985896; doi:10.7717/peerj.14918)
Supplement: Supplemental Information 9 [file peerj-11-14918-s009.docx]

**Table S5: Relative gene expression fold change of CD86 mRNAs in different iMΦ groups.**

| **Group** | **Expression Fold Change of CD86** | |
| --- | --- | --- |
| Mock | 1.39 | 0.72 |
| Delta  24 HPI | 2.92 | 3.15 |
| Delta  48 HPI | 4.01 | 1.67 |
| Delta  72 HPI | 5.68 | 4.13 |
| Omicron  24 HPI | 3.40 | 3.20 |
| Omicron  48 HPI | 3.72 | 4.13 |
| Omicron  72 HPI | 7.83 | 6.88 |
| LPS/IFNγ | 0.91 | 0.37 |
